# Neuroprotective effect of resveratrol on Epac-1/Rap-1 signaling pathway in ischemic stroke rats

**DOI:** 10.3389/fnins.2025.1703951

**Published:** 2026-01-12

**Authors:** Jinrong Li, Jianjun He, Kunli Gao, Yue Zou

**Affiliations:** 1 Health Management Center, The Fourth Affiliated Hospital of Southwest Medical University, Meishan, Sichuan, China; 2 Department of General Surgery, Affiliated Hospital of Traditional Chinese Medicine of Southwest Medical University, Luzhou, Sichuan, China; 3 Department of General Practice, Affiliated Hospital of Southwest Medical University, Luzhou, Sichuan, China

**Keywords:** cerebral infarction, Epac-1/Rap-1 signaling pathway, ischemia–reperfusion injury, neuroprotection, resveratrol

## Abstract

**Objective:**

To evaluate the neuroprotective effect of resveratrol (Res) and elucidate its underlying mechanism in a rat model of cerebral ischemia–reperfusion injury.

**Methods:**

Adult male Sprague–Dawley rats received intraperitoneal Res (40 mg/kg/day) for three consecutive days, followed by transient middle cerebral artery occlusion/reperfusion (MCAO/R). Animals were randomly divided into Sham, MCAO/R, and Res groups. Neurological function was evaluated 24 h after reperfusion. Cerebral infarct volume was assessed by 2,3,5-triphenyltetrazolium chloride (TTC) staining. The serum levels of M1/M2 polarization markers, including the enzymes inducible nitric oxide synthase (iNOS) and arginase-1 (Arg-1), as well as the cytokines interleukin-12 (IL-12) and interleukin-10 (IL-10), were measured by enzyme-linked immunosorbent assay (ELISA). Apoptosis (non-specific neuronal apoptosis) was detected by the terminal deoxynucleotidyl transferase dUTP nick-end labeling (TUNEL) staining method. To investigate the underlying mechanism, we focused on the exchange protein directly activated by cAMP-1 (Epac-1) and its downstream effector Ras-related protein 1 (Rap-1), which are known regulators of neuroinflammation. The expression levels of the exchange proteins directly activated by Epac-1 and Rap-1 in ischemic brain tissue were detected using Western blotting.

**Results:**

Relative to the Sham group, the MCAO/R group exhibited significantly larger infarct volumes and higher neurological deficit scores, together with increased serum iNOS and IL-12 and decreased IL-10 and Arg-1 (all *p* < 0.05). The apoptosis of neuronal cells increases while the expressions of Epac-1 and Rap-1 proteins decrease (*p* < 0.05). Res treatment significantly reduced infarct size and neurological deficits; lowered serum iNOS and IL-12; raised IL-10 and Arg-1; and improved Epac-1 and Rap-1 expression compared with the MCAO/R group (all *p* < 0.05).

**Conclusion:**

Res exerts neuroprotective effects against cerebral ischemia–reperfusion injury, potentially by modulating microglial polarization toward the M2 phenotype via the Epac-1/Rap-1 signaling pathway, thereby suppressing inflammation and mitigating neuronal damage.

## Introduction

Cerebral ischemic stroke (IS) remains one of the leading causes of mortality and disability worldwide, with its incidence steadily rising and showing a concerning trend toward younger onset (GBD 2021 Nervous System Disorders Collaborators, 2024; He et al., 2025). Approximately 87% of all strokes are ischemic stroke (IS) in nature (Tsao et al., 2023). The current cornerstone of acute IS treatment involves rapid restoration of blood flow through intravenous thrombolysis and/or mechanical thrombectomy. However, the clinical utility of these interventions is severely constrained by a narrow therapeutic window and the inherent risk of hemorrhagic complications (Zhang et al., 2024). Crucially, the process of reperfusion itself can paradoxically induce additional brain damage, known as cerebral ischemia–reperfusion injury (CIRI), which exacerbates the initial ischemic insult and contributes to poor neurological outcomes (Wang et al., 2022). The pathophysiology of CIRI is complex and multifactorial, involving a cascade of interconnected events such as oxidative stress, calcium overload, excitotoxicity, mitochondrial dysfunction, and robust neuroinflammation (Wang et al., 2022; Fisher and Savitz, 2022).

Among these mechanisms, post-ischemic inflammation plays a pivotal role in both the progression and long-term outcomes of CIRI (Yuan et al., 2021). Within hours of ischemia, the brain’s resident immune cells, microglia, become rapidly activated (Xu et al., 2020). These cells exhibit a high degree of plasticity and can polarize into distinct functional phenotypes in response to local signals. The classical M1 phenotype is pro-inflammatory, releasing cytotoxic mediators like reactive oxygen species and pro-inflammatory cytokines, thereby exacerbating neuronal injury. In contrast, the alternative M2 phenotype is anti-inflammatory and reparative, promoting tissue repair through the secretion of factors like IL-10 and Arg-1, which facilitate neurogenesis and debris clearance (Lian et al., 2020; Ma et al., 2021). Hence, modulating the M1/M2 polarization balance may represent a promising therapeutic strategy for mitigating CIRI.

Among the diverse intracellular signaling cascades implicated in neuroinflammation, the cAMP-activated exchange protein directly activated by cAMP 1 (Epac-1) and its downstream effector Ras-related protein 1 (Rap-1) have gained increasing attention. The Epac-1/Rap-1 pathway plays a key role in regulating endothelial integrity, oxidative stress, immune cell activation, and inflammatory cytokine release (Virwani et al., 2022; Garcia-Morales et al., 2017; El-Mokadem et al., 2021). Recent studies have shown that Epac-1 activation can reduce neuronal apoptosis, attenuate blood–brain barrier disruption, and modulate immune responses in various models of ischemic injury (El-Mokadem et al., 2021; Sun et al., 2023). Moreover, Epac-1/Rap-1 signaling has been implicated in microglial function and phenotype switching, suggesting it may serve as a novel therapeutic target in cerebral ischemia–reperfusion injury (Sun et al., 2023; Pan et al., 2022).

Given these challenges, there is growing interest in identifying novel therapeutic agents for CIRI. Traditional Chinese medicine (TCM), known for its affordability, safety, and efficacy, offers a valuable resource for drug discovery. Several TCM compounds have demonstrated neuroprotective effects in models of acute ischemic stroke. Resveratrol (3,4′,5-trihydroxy-stilbene, Res), a natural polyphenol abundant in grapes, berries, and peanuts, has garnered considerable attention for its pleiotropic pharmacological properties (Katila et al., 2022; Ren et al., 2021; Berretta et al., 2020). Although Res has been extensively studied in the context of CIRI (Lopez-Morales et al., 2023), its potential role in modulating microglial polarization remains unexplored.

## Materials and methods

### Grouping and administration of experimental animals

Thirty healthy adult male SPF-grade Sprague–Dawley (SD) rats (6–8 weeks old) were purchased from Spiff (Beijing) Biotechnology Co., Ltd. [SCXY(Jing) 2024-0001]. The animals were housed at a controlled room temperature of 26 ± 2 °C and relative humidity of 50–60%, under a 12 h light/dark cycle, with free access to food and water. After 1 week of adaptation, the rats were randomly divided into three groups (*n* = 10): Sham group, MCAO/R group and MCAO/R-Res (Res) group. The Sham group and the middle cerebral artery occlusion/reperfusion (MCAO/R) group received daily intraperitoneal injections of normal saline at 8:00 a.m. for 3 consecutive days before surgery; the Res group received intraperitoneal injection of Res (40 mg/kg, dissolved in DSMO) on the same schedule.

After drug administration, MCAO/R models were established in the MCAO/R and Res groups using the intraluminal thread occlusion method as described in previous studies (Sun et al., 2023; Hu et al., 2022). Rats were anesthetized by intraperitoneal injection of 3% pentobarbital sodium (30 mg/kg) and placed in a prone position on the operating table with limbs secured. A midline neck incision was made, the thyroid gland was bluntly dissected and retracted, and a paperclip bent into a hook shape was used to separate the internal carotid artery (ICA), external carotid artery (ECA), and right common carotid artery (CCA). The CCA and ECA were ligated with sutures, and the ICA was temporarily clamped. A nylon filament was then inserted through the CCA into the middle cerebral artery and secured in place. The muscle and skin were sutured, taking care to leave about 1 cm of filament protruding from the incision. After 2 h of occlusion, the filament was removed to allow reperfusion. All animal experimental procedures were reviewed and approved by the Laboratory Animal Welfare & Ethics Committee of Bestcell Model Biological Center [Ethical Approval No. 2024-04-18B], and were conducted in accordance with its institutional guidelines.

Although 10 rats were allocated to each group at the beginning of the study, not all animals survived MCAO/R surgery. A total of six rats per group completed all procedures and were included in the final analyses.

### Neurological deficit score

The improved Bederson 5-point scoring method (Bederson et al., 1986) was adopted for evaluation: 0 score was normal: no motor defect; 1 was mild, cannot extend the healthy side of the front paw when lifting the tail; 2 divided into moderate, turn to the healthy side; 3 can be classified as severe, unable to move to the healthy side; 4 was classified as extremely severe, involuntary movement, and decreased consciousness.

### 2,3,5-Triphenyltetrazolium chloride staining (TTC staining)

After the modeling procedure, the brain of the rats were harvested and placed in a − 20 °C freezer for 20 min for rapid freezing. Subsequently, coronal brain sections were prepared and incubated in 2% TTC solution at 37 °C for 30 min in the dark (wrapped in tin foil). Brain slices showing clear staining were laid flat and photographed using a digital camera. Image analysis software was used to analyze the infarct areas and calculate infarct volume.

### TUNEL staining was used to detect apoptosis in brain tissue of rats

Brain tissue samples were removed from liquid nitrogen storage, fixed in 4% paraformaldehyde, dehydrated, paraffin-embedded, and sectioned. Tissue sections were deparaffinized and rehydrated by sequential immersion in xylene I and II, followed by anhydrous ethanol I and II, 95, 85, and 75% ethanol, for 5 min each. Sections were then rinsed with PBS three times. Next, 50 μL of proteinase K solution was added to each section, followed by incubation at 37 °C for 30 min, and rinsing with phosphate-buffered saline (PBS, 3 times). Then, 50 μL of sodium citrate solution was added, incubated at room temperature for 4 min, and washed again with PBS (3 times). TUNEL staining was performed according to the manufacturer’s instructions. TUNEL reaction solution was applied and incubated at 37 °C for 60 min, followed by three PBS washes. A POD converter solution was added and incubated at 37 °C for 30 min, then washed with PBS three times. Color development was performed using a DAB staining kit, followed by three PBS washes. Slides were counterstained with hematoxylin for 60 s and rinsed with running water for 20 min. Differentiation was performed in 1% hydrochloric acid-alcohol solution for 1–2 s, followed by rinsing under running water for another 20 min. Finally, the sections were coverslipped. Apoptotic neurons were visualized as green fluorescence signals, and nucleus were counterstained with blue fluorescence.

### Hematoxylin–eosin staining

Paraffin-embedded brain tissue sections were baked in a 65 °C oven for 2 h, followed by dewaxing in xylene I, II, and III for 10 min each. After gradient ethanol hydration, HE staining was performed, followed by dehydration with graded ethanol, clearing with xylene, and coverslipping. The ischemic penumbra surrounding the infarct core was examined under a light microscope at 400 × magnification. Representative fields were selected, and histopathological changes in brain tissue were evaluated across different groups.

### Enzyme-linked immunosorbent assay

Serum samples were collected from anesthetized rats via cardiac puncture, allowed to clot at room temperature, and centrifuged at 3000 rpm for 15 min to obtain supernatants. The concentrations of inducible nitric oxide synthase (iNOS), arginase-1 (Arg-1), interleukin-12 (IL-12), and interleukin-10 (IL-10) were measured using ELISA kits (mlbio, Shanghai), following the manufacturer’s instructions. Briefly, 100 μL of standards or diluted serum samples were added to each well of a 96-well microplate pre-coated with specific antibodies, and incubated at 37 °C for 1 h. Wells were then washed 3–5 times with washing buffer, followed by the addition of 100 μL of biotinylated detection antibody to each well and further incubation at 37 °C for 30 min. After washing, 100 μL of HRP-conjugated streptavidin was added and incubated for another 30 min at 37 °C.

After a final washing step, 100 μL of TMB substrate solution was added to each well and incubated in the dark for 10–15 min. The reaction was stopped by adding 50 μL of stop solution, and absorbance was measured at 450 nm using a microplate reader. Cytokine concentrations were calculated based on the standard curves generated from known concentrations of standards using curve-fitting software.

### Western blot analysis

The tissues were rinsed 3 times with pre-cooled PBS to remove blood residues, cut into small pieces, and homogenates were transferred to the centrifuge tubes, shaken, and kept on ice for 30 min. During this time, samples were intermittently pipetted to ensure complete cell lysis. The lysates were then centrifuged at 12,000 g for 5 min at 4 °C, and the supernatants were collected. The total protein concentration was determined using a bicinchoninic acid (BCA) protein assay kit (Beyotime, China). Equal amounts of protein were mixed with 5 × loading buffer, boiled at 95 °C for 10 min, and subjected to SDS-PAGE using 5% stacking gel and 12% separating gel. Proteins were then transferred onto polyvinylidene difluoride (PVDF) membranes (Millipore, United States) using a semi-dry transfer method. After blocking with 5% non-fat milk in TBST for 1 h at room temperature, the membranes were incubated overnight at 4 °C with primary antibodies: anti-GAPDH (Abcam, ab128915, 1:10000); anti-Epac-1 (Abcam, ab109415, 1:1000) and anti-Rap-1 (Abcam, ab14404, 1:1000). The next day, membranes were washed three times with 1 × TBST (5 min each), followed by incubation with HRP-conjugated secondary antibody (goat anti-mouse IgG, 1:5000, Affinity, S0001) at room temperature for 30 min. Membranes were washed four times (10 min each), and signals were visualized using an enhanced chemiluminescence (ECL) kit (Beyotime, China). Protein bands were detected and analyzed using image acquisition software, the protein expression levels of Epac-1 and Rap-1 were normalized to that of GAPDH, which served as the internal control protein and membranes were scanned and archived.

### Statistical analysis

Statistical analysis was performed using SPSS software (version 23, IBM Corp., United States). Data were expressed as mean ± standard deviation (SD). The Shapiro–Wilk test was used to assess the normality of data distribution. For data that met normal distribution and homogeneity of variance, one-way analysis of variance (one-way ANOVA) was applied to compare differences among multiple groups, followed by Tukey’s honestly significant difference (HSD) *post hoc* test for pairwise comparisons. For comparisons between two groups, an independent-samples *t*-test was used. The neurological deficit score was expressed as the median (Q1, Q3) and non-parametric Kruskal-Wallis H test was employed for comparisons among multiple groups, followed by Dunn’s post-hoc test for pairwise comparisons between groups. A *p*-value < 0.05 was considered statistically significant.

## Results

### Effect of resveratrol on neural function deficit score of rats in each group

**Figure 1:**
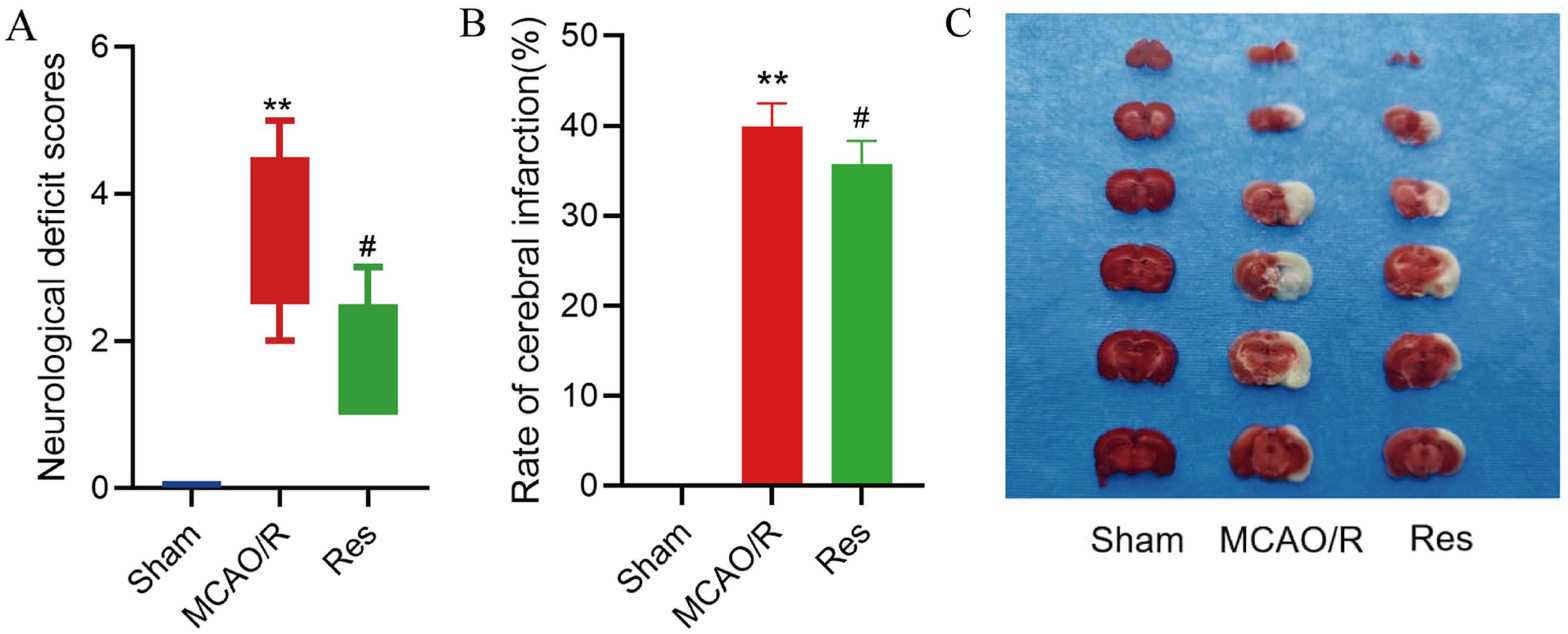
Resveratrol (Res) alleviates neurological deficits and infarction after cerebral I/R injury. **(A)** The neurological deficit score 24 h after reperfusion. The Kruskal-Wallis test was used for analysis, followed by Dunn’s *post hoc* test. **(B)** Quantitative cerebral infarction volume (as a percentage of the cerebral hemisphere) stained with TTC. The data are presented as mean ± SD and analyzed using one-way ANOVA and Tukey’s post hoc test. **(C)** Representative TTC-stained brain sections of the Sham, MCAO/R, and Res groups. *n* = 6. The data were analyzed using non-parametric tests. ** *p* < 0.01 vs. Sham; # *p* < 0.05 vs. MCAO/R. MCAO/R, middle cerebral artery occlusion/reperfusion; TTC, 2,3,5-triphenyltetrazolium chloride; I/R, ischemia/reperfusion.

At 24 h after ischemia–reperfusion, the neurological deficit score was significantly increased in the MCAO/R group compared with the Sham group (*p* < 0.01). However, Res treatment markedly reduced the neurological deficit score compared with the MCAO/R group (*p* < 0.05) (Figure 1A).

### Effect of resveratrol on cerebral infarction rate of rats in each group

At 24 h after ischemia–reperfusion, the cerebral infarction rate in the MCAO/R group was significantly higher than that in the Sham group (*p* < 0.01). Moreover, the infarct rate in the Res group was significantly reduced compared with the MCAO/R group (*p* < 0.05), which indicated a neuroprotective effect of Res treatment (Figure 1B). The cerebral infarction tissue sections of MCAO/R group and Res group showed white infarcts, and the infarct volume of Res group was significantly reduced compared with that of MCAO/R group (Figure 1C).

### Effect of resveratrol on pathological changes in rat brain tissue

**Figure 2:**
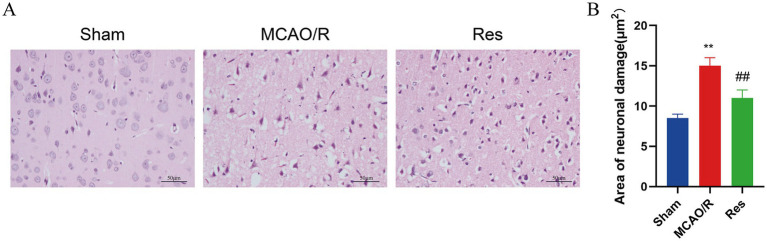
Histological changes in brain tissue assessed by HE staining. **(A)** Representative H&E-stained brain sections from the Sham group, the MCAO/R group, and the Res group (scale = 50 μm). **(B)** Quantitative assessment of neuronal damage based on HE staining *n* = 6. The data were analyzed using Tukey’s honestly significant difference (HSD) test. ** *p* < 0.01 vs. Sham; ## *p* < 0.01 vs. MCAO/R. HE, hematoxylin–eosin.

Hematoxylin–eosin staining revealed that the brain structure in the Sham group was intact, with neatly arranged neurons, clearly defined nucleus, and no evidence of interstitial edema. In contrast, the MCAO/R group exhibited significant pathological damage, including disorganized neuronal arrangement, nuclear pyknosis and karyolysis, interstitial loosening, and cytoplasmic vacuolization. Notably, the Res group showed improved brain tissue morphology compared with the MCAO/R group, characterized by more orderly cell arrangement and more intact nuclei (Figure 2A). Quantitative analysis showed that compared with the MCAO/R group, the number of damaged neurons in the Res group was significantly reduced (*p* < 0.05) (Figure 2B), which indicates that resveratrol has neuroprotective effects.

### Effect of resveratrol on apoptosis rate of rat brain tissue cells in each group

**Figure 3:**
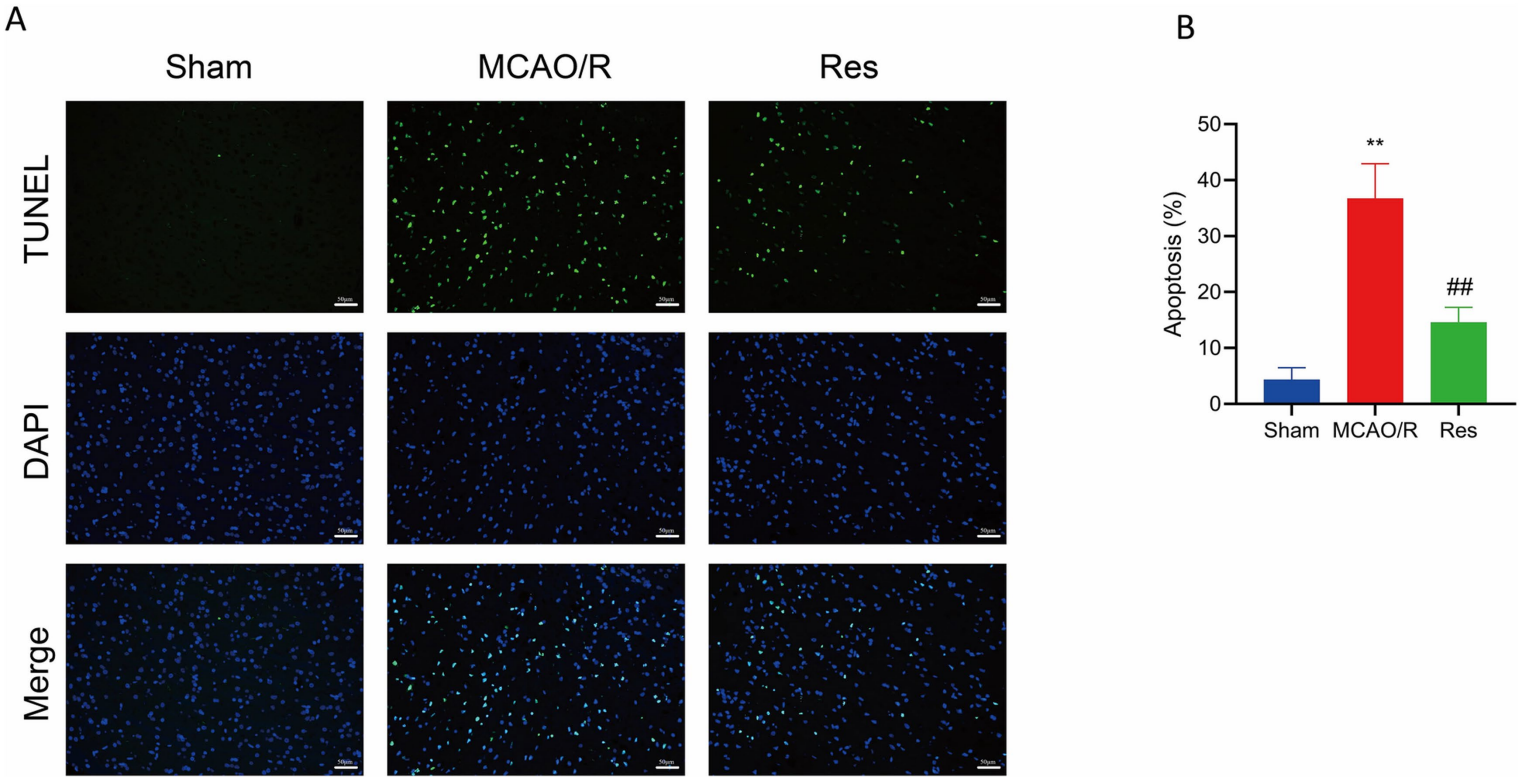
Effect of Res on neuronal apoptosis after cerebral ischemia–reperfusion injury. **(A)** Representative TUNEL staining images from the Sham, MCAO/R, and Res groups. Apoptotic cells (green) were visualized by TUNEL staining; nucleus were counterstained with DAPI (blue). Scale bar = 50 μm. **(B)** Quantification of apoptotic cells. *n* = 6. The data were analyzed using Tukey’s honestly significant difference (HSD) test. * *p* < 0.01 vs. Sham; ## *p* < 0.01 vs. MCAO/R. TUNEL, terminal deoxynucleotidyl transferase dUTP nick end labeling; DAPI, 4′,6-diamidino-2-phenylindole.

TUNEL staining was used to detect apoptotic cells in the surrounding tissues of cerebral infarction, with green fluorescence indicating TUNEL-positive (apoptotic) cells. No green fluorescence was observed in the Sham group, suggesting the absence of apoptosis. In contrast, the MCAO/R group showed extensive green fluorescence, indicating a high level of neuronal apoptosis. The Res group exhibited a marked reduction in green fluorescence intensity compared with the MCAO/R group, which indicated fewer apoptotic cells (Figure 3A). Quantitative analysis further confirmed that the level of apoptosis was significantly increased in the MCAO/R group compared with the Sham group, whereas Res treatment significantly reduced apoptosis levels (Figure 3B).

### Effects of resveratrol on levels of iNOS, Arg-1 and inflammatory factors IL-12 and IL-10 in serum of rats in each group

**Figure 4:**
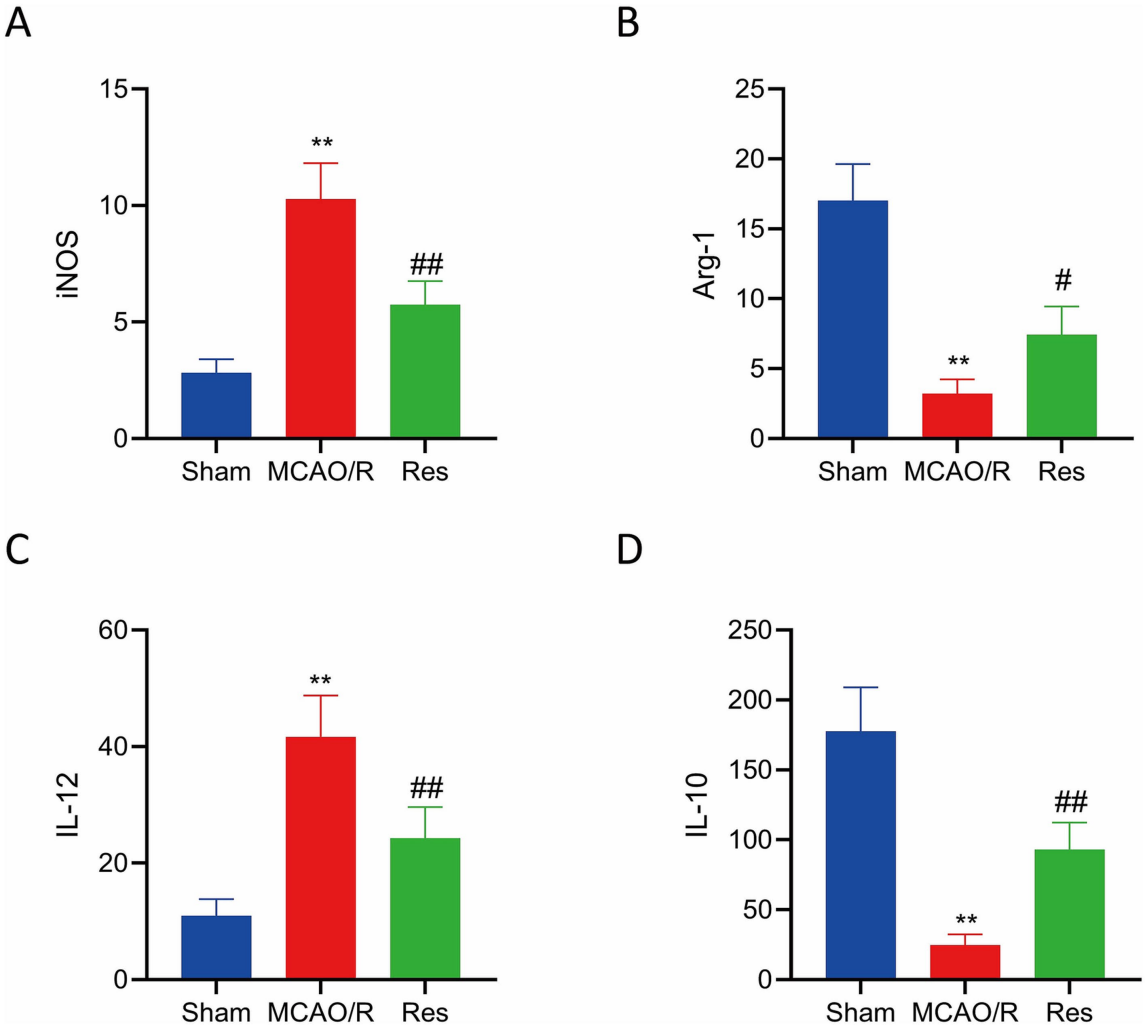
Res modulates serum inflammatory markers after cerebral I/R. **(A)** Expression of iNOS. **(B)** Expression of Arg-1. **(C)** Levels of IL-12. **(D)** Levels of IL-10. *n* = 6. The data were analyzed using Tukey’s honestly significant difference (HSD) test. ** *p* < 0.01 vs. Sham; # *p* < 0.05, ## *p* < 0.01 vs. MCAO/R. iNOS, inducible nitric oxide synthase; Arg-1, arginase-1; IL, interleukin.

Compared with the sham operation group, the serum levels of M1-type markers iNOS and cytokine IL-12 were significantly increased in the MCAO/R group and the Res group (*p* < 0.05), while the levels of M2-type marker Arg-1 and cytokine IL-10 were significantly decreased (*p* < 0.05). However, compared with the MCAO/R group, the Res group showed significantly lower levels of iNOS and IL-12 (*p* < 0.05), and significantly higher levels of IL-10 and Arg-1 (*p* < 0.05), indicating that Res may promote M2 polarization and suppress M1 polarization by modulating the expression of these cytokines (Figures 4A–D).

### Effects of resveratrol on the expression levels of Epac-1 and Rap-1 proteins in brain tissue of rats in each group

**Figure 5:**
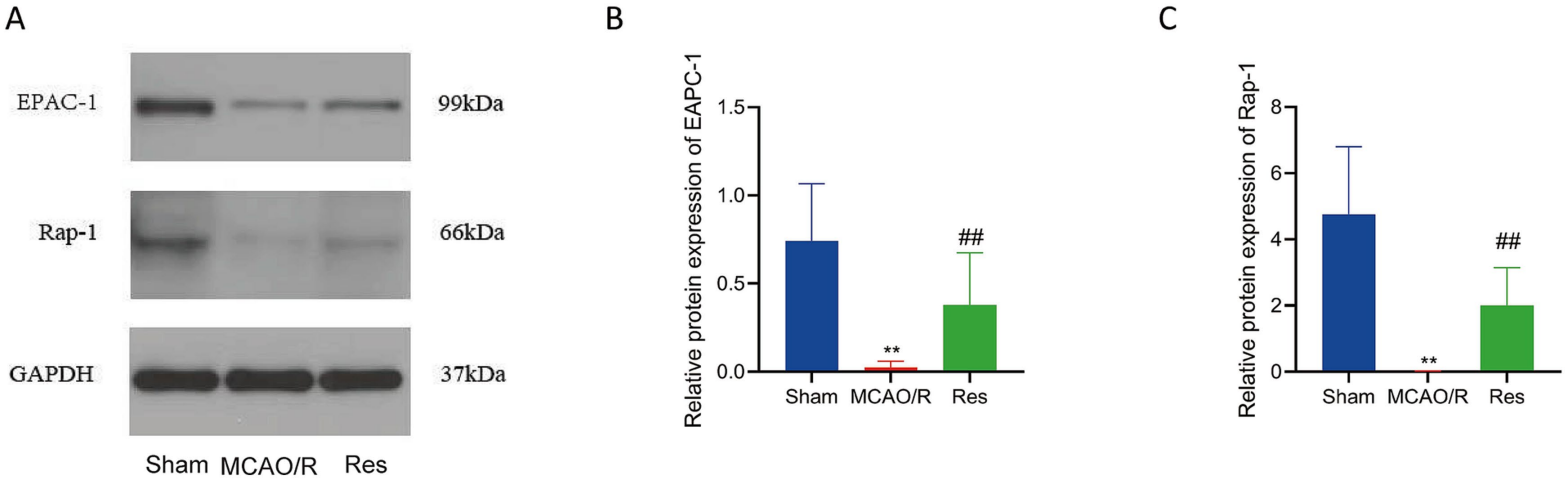
Effects of Res on Epac-1 and Rap-1 protein expression in brain tissue. **(A)** Representative Western blot bands showing Epac-1 and Rap-1 expression in the Sham, MCAO/R, and Res (Res) groups; GAPDH was used as a loading control. **(B,C)** Quantitative analysis of Epac-1 **(B)** and Rap-1 **(C)** relative protein expression. *n* = 6. The data were analyzed using Tukey’s honestly significant difference (HSD) test. ** *p* < 0.01 vs. Sham; ##*p* < 0.01 vs. MCAO/R. Epac-1, exchange protein directly activated by cAMP 1; Rap-1, Ras-related protein 1.

Western blot results showed that the protein expression levels of Epac-1 and Rap-1 in the brain tissue of rats in the MCAO/R group were significantly decreased compared with those in the Sham group (*p* < 0.01). After treatment with Res, the expression levels of both proteins were significantly increased compared with the MCAO/R group (*p* < 0.01) (Figures 5A–C), which suggested that the Epac-1/Rap-1 signaling pathway was suppressed during MCAO/R injury and could be partially restored by Res intervention.

**Figure 6:**
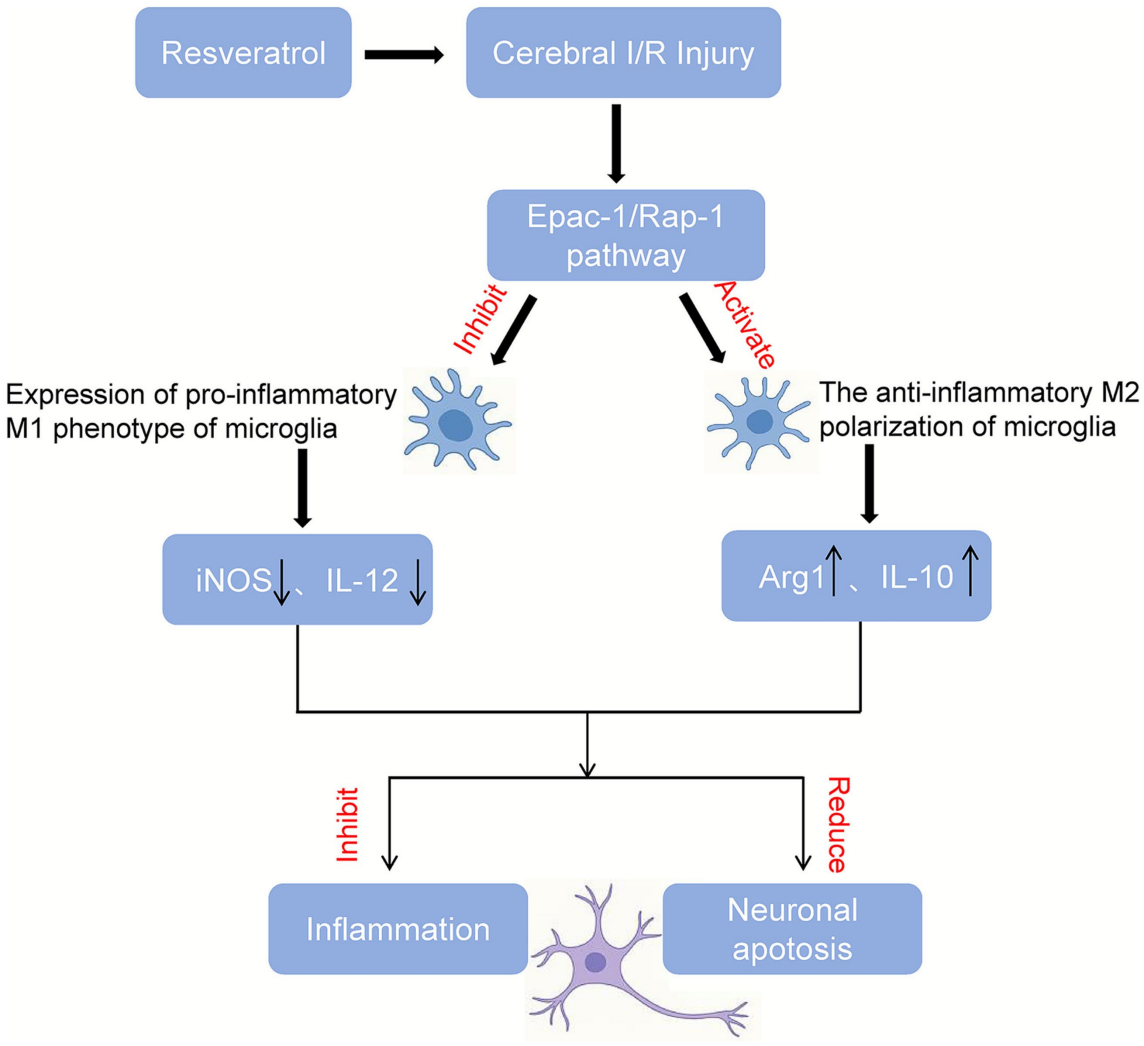
Proposed mechanism of resveratrol-mediated neuroprotection through the Epac-1/Rap-1 signaling pathway.

To further illustrate the downstream mechanism underlying these findings, a schematic diagram of the proposed Epac-1/Rap-1-mediated neuroprotective pathway is presented in Figure 6.

## Discussion

Res, a natural polyphenolic compound, was first isolated from Veratrum grandiflorum by Takaoka in 1939 (Breuss et al., 2019). Numerous studies have demonstrated that long-term use of Res significantly reduces reactive oxygen species production and lipid peroxidation, while enhancing antioxidant enzyme activities, thereby exerting strong antioxidant and anti-inflammatory effects (Juarez et al., 2023). In a diabetic rat model of cerebral ischemia–reperfusion, administration of Res at a dose of 20 mg/kg effectively reduced levels of malondialdehyde (MDA), TNF-*α*, IL-6 and myeloperoxidase (MPO), while increasing levels of catalase (CAT), superoxide dismutase (SOD), and IL-10 (Prabhakar, 2013). Additionally, Res significantly increased the expression of the Bcl-2 and decreased the expression of Bax, cleaved caspase-3 and total caspase-3, indicating that it may alleviated cerebral ischemia reperfusion injury by inhibiting neuronal apoptosis (Fang et al., 2015). Our results are consistent with these findings. Compared with the MCAO/R group, rats in the Res group exhibited more intact brain tissue structure, clearer nuclear morphology, fewer cells with karyolysis and pyknosis, reduced neurological deficit scores, smaller infarct volumes, and decreased neuronal apoptosis. Microglia, the resident immune cells of the central nervous system, play a central role in the neuroinflammatory response associated with acute brain injury and neurodegenerative diseases (Kerr et al., 2019; Iadecola and Anrather, 2011). As the first line of defense against ischemic stroke, microglia are key participants in its pathogenesis (Jia et al., 2021). These cells exhibit high plasticity and can polarize into two major phenotypes in response to environmental cues: the classically activated pro-inflammatory M1 type and the alternatively activated anti-inflammatory M2 type (Hu, 2020). M1-type microglia predominate in the peri-infarct region and are characterized by the production of inflammatory cytokines (e.g., IL-1*β*, IL-6, TNF-α, IFN-*γ*), neurotoxic mediators (e.g., nitric oxide, ROS, MMPs, prostaglandins), and the marker iNOS (Li et al., 2022). In contrast, M2-type microglia dominate the infarct core region and contribute to tissue repair via secretion of anti-inflammatory cytokines (e.g., IL-4, IL-10, TGF-β, IGF-1) and the expression of Arginase-1 (Arg-1), a marker associated with nitrogen metabolism and neuroprotection (Li et al., 2022). However, M2 function is transient and fades within 7 days post-injury, while M1 activation typically peaks within 2 weeks, exacerbating inflammation and injury (Luo et al., 2022). Given this dynamic, recent studies have suggested that promoting the transition from M1 to M2 microglia or inhibiting M2-to-M1 conversion in the early stages of ischemic injury may provide a neuroprotective strategy for stroke intervention (Kim et al., 2016; Lan et al., 2017). Our data demonstrate that Res administration significantly altered the balance of microglial polarization markers, decreasing serum levels of the M1-associated enzyme iNOS and the cytokine IL-12, while increasing the M2-associated cytokine IL-10 and the enzyme Arg-1.

Mechanistically, we focused on the Epac-1/Rap-1 signaling pathway, a recently identified branch of the cAMP signaling cascade. Epac-1 (Exchange Protein Directly Activated by cAMP 1) is a guanine nucleotide exchange factor (GEF) that activates the small GTPase Rap1 by facilitating GDP-GTP exchange (Wehbe et al., 2020; de Rooij et al., 2000). This pathway regulates a variety of cellular functions, including neuronal excitability, axonal guidance, cell adhesion, and inflammation modulation (Schmidt et al., 2013; Robichaux and Cheng, 2018). Recent studies have shown that the Epac-1/Rap-1 axis plays a protective role in ischemia–reperfusion injury. For example, Vitexin was shown to ameliorate myocardial injury via this pathway (Yang et al., 2021), and activation of EPAC signaling has been linked to protection of the blood–brain barrier after ischemic stroke (Chu et al., 2021).

Recent evidence also suggests that the Epac-1/Rap-1 axis may participate directly in microglial phenotype regulation. Although research on microglia is still limited, the activation of Epac-1 has been proven to inhibit NF-κB-dependent inflammatory responses and reduce oxidative stress, which is the main driving factor for M1 polarization (Gerlo et al., 2011; Parnell et al., 2012). In macrophages closely related to microglia—the activation of Epac-1 promotes the transformation to an anti-inflammatory phenotype (Wiejak et al., 2024), and Rap1 can regulate the inflammatory signal transduction pathway to induce the functional activation of integrin αMβ2 in macrophages, suggesting that the Epac-1/Rap proteins are closely related to macrophages (Caron et al., 2000). In macrophages, the Epac-1/Rap protein is closely associated with macrophages. In the context of cerebral ischemia–reperfusion injury, Epac-1/Rap-1 signaling has been shown to reduce blood–brain barrier disruption, immune activation, and neuronal damage (Sun et al., 2023). Our findings that resveratrol restores Epac-1 and Rap-1 expression while simultaneously decreasing iNOS/IL-12 and increasing Arg-1/IL-10 suggest that Epac-1/Rap-1 reactivation may facilitate a shift toward M2-type microglial polarization. However, the downstream molecular intermediates linking Epac-1/Rap-1 to microglial phenotype switching remain insufficiently defined, and further mechanistic studies are warranted.

Several limitations of this study should be acknowledged. First, resveratrol is dissolved in dimethyl sulfoxide (DMSO). It should be noted that DMSO may have a brain-protective effect (Shimizu et al., 1997), and we cannot rule out the potential interference effect of DMSO in the Res treatment group. Future research should set up a control group with a carrier (using only DMSO) to more clearly demonstrate the effect of Res. Secondly, Cerebral blood flow was not directly monitored using laser-Doppler flowmetry during occlusion or reperfusion. Although model success was evaluated based on standard surgical criteria, the lack of real-time perfusion monitoring may introduce variability. Future studies should incorporate laser-Doppler flowmetry to more accurately confirm ischemia and reperfusion. Thirdly, there are currently few studies on the role of the Epac1/Rap1 signaling pathway in promoting the M1/M2 anti-inflammatory polarization of microglia. The potential effects of this pathway deserve further exploration. The specific downstream events indicated by Epac-1/Rap-1 signal transduction for microglial polarization still need to be further clarified. Finally, although Res treatment restored Epac-1 and Rap-1 expression, the current data cannot distinguish whether this effect is due to direct activation of the Epac-1/Rap-1 pathway or an indirect consequence of reduced neuroinflammation and tissue injury. Future studies employing Epac-specific modulators and Rap1 activation assays will be necessary to determine the mechanistic basis of this regulation.

## Conclusion

Our research indicates that resveratrol is an effective neuroprotectant in rat models of ischemic stroke. It can restore the function of the inhibited Epac-1/Rap-1 signaling pathway, cause microglia to polarize toward M2, alleviate neuroinflammation and cell apoptosis, and ultimately improve neurological function. These findings not only deepen our understanding of the mechanism of action of resveratrol, but also highlight the potential of the Epac-1/Rap-1 pathway as a therapeutic target for ischemic stroke.

## Data Availability

The datasets used and/or analyzed during the current study are available from the corresponding author on reasonable request.
